# Successful Percutaneous Coronary Intervention for Chronic Total Occlusion in Left Ventricular Systolic Dysfunction Patients with and without Diabetes Mellitus

**DOI:** 10.31083/j.rcm2511396

**Published:** 2024-11-07

**Authors:** Xi Wu, Qin Li, Mingxing Wu, Haobo Huang, Zhe Liu, He Huang, Lei Wang

**Affiliations:** ^1^Department of Cardiology, Xiangtan Central Hospital, 411100 Xiangtan, Hunan, China

**Keywords:** chronic total occlusion, percutaneous coronary intervention, diabetes mellitus, major adverse cardiac events, left ventricular systolic dysfunction

## Abstract

**Background:**

Diabetes mellitus (DM) and left ventricular (LV) systolic dysfunction are common in patients who receive percutaneous coronary intervention (PCI) for chronic total occlusion (CTO). This study aimed to investigate the clinical outcomes of LV systolic dysfunction patients who had successful PCI for CTO over two years, with or without DM.

**Methods:**

This cohort included 185 patients with LV systolic dysfunction undergoing successful PCI for CTO. A comparative analysis was performed on individual data and clinical outcomes among patients with and without DM after a two-year follow-up.

**Results:**

DM was identified in 99 (53.5%) patients who exhibited a higher incidence of chronic kidney disease (CKD), elevated serum creatinine levels, increased hemoglobin A1c, and reduced estimated glomerular filtration rates (*p* < 0.05). Patients with diabetes also experienced increased multi-vessel disease, a higher number of lesions per patient, as well as elevated multicenter chronic total occlusion registry in Japan (J-CTO) and Synergy between Percutaneous Coronary Intervention with Taxus and Cardiac Surgery (SYNTAX) scores (*p* < 0.05). During the two-year follow-up, the DM group showed a greater occurrence of major adverse cardiovascular events (MACEs) compared with the non-DM group (24.2% versus 12.8%, *p* < 0.001). The DM group also had higher rates of all-cause mortality (9.1% versus 3.5%, *p* < 0.002), cardiac death (8.1% versus 1.2%, *p* < 0.001), and target vessel revascularization (18.2% versus 7.1%, *p* < 0.001). Multivariable logistic regression analysis demonstrated that the presence of DM is not an independent predictor of MACEs (hazard ratio (HR): 0.58; 95% confidence interval (CI): 0.32 to 1.03; *p* = 0.260). Moreover, the multi-vessel disease (HR: 1.69; 95% CI: 1.21 to 2.36; *p* = 0.002), CKD (HR: 1.38; 95% CI: 1.08 to 1.78; *p* = 0.011) and complete revascularization (HR: 0.36; 95% CI: 0.14 to 0.88; *p* = 0.026) had a significant association with MACEs.

**Conclusions:**

In patients with LV systolic dysfunction who underwent successful CTO-PCI, those with diabetes exhibited a higher trend toward the incidence of MACEs over two years.

## 1. Introduction

Approximately 30%–50% of individuals receiving coronary angiography with the diagnosis of coronary artery disease (CAD) exhibit chronic total occlusion (CTO) [1]. Previous retrospective research has shown that effective percutaneous coronary intervention (PCI) for CTO can improve ventricular function and decrease the symptoms of angina and dyspnea compared to failed attempts at revascularization [2, 3]. Studies have shown that diabetes mellitus (DM) is common in patients with CAD [4] and a risk factor for the occurrence of CTO [5, 6]. Research also revealed that 27% to 45% of patients undergoing CTO revascularization have DM [5, 7, 8]. Currently, there is controversy regarding the effect of DM on clinical results after successful revascularization with PCI. The research findings are still controversial: some studies have reported that the incidence of major adverse cardiac events (MACEs) is higher in diabetic patients [8, 9, 10], whereas others have observed no significant differences in MACE rates between diabetic and non-diabetic patients [11, 12, 13]. Left ventricular ejection fraction (LVEF) is an important predictor of cardiovascular events in CAD patients [14]. Evidence suggests that in patients with reduced LVEF, the presence of CTO correlates with poorer clinical outcomes [14]. However, CTO-PCI can potentially relieve angina symptoms in these patients and also improve LVEF in carefully selected cases [15].

Many physicians in clinical practice hesitate to undertake PCI for CTO lesions in patients suffering from left ventricular (LV) systolic dysfunction and DM because of concerns about safety during the procedure and unknown long-term benefits. Insufficient data exists concerning the long-term clinical impacts of successful PCI for CTO on LV systolic dysfunction in patients with DM. This study sought to assess the clinical results for successful PCI for CTO in individuals with LV systolic dysfunction over two years.

## 2. Material and Methods

### 2.1 Study Design and Patients

**Fig. 1.**
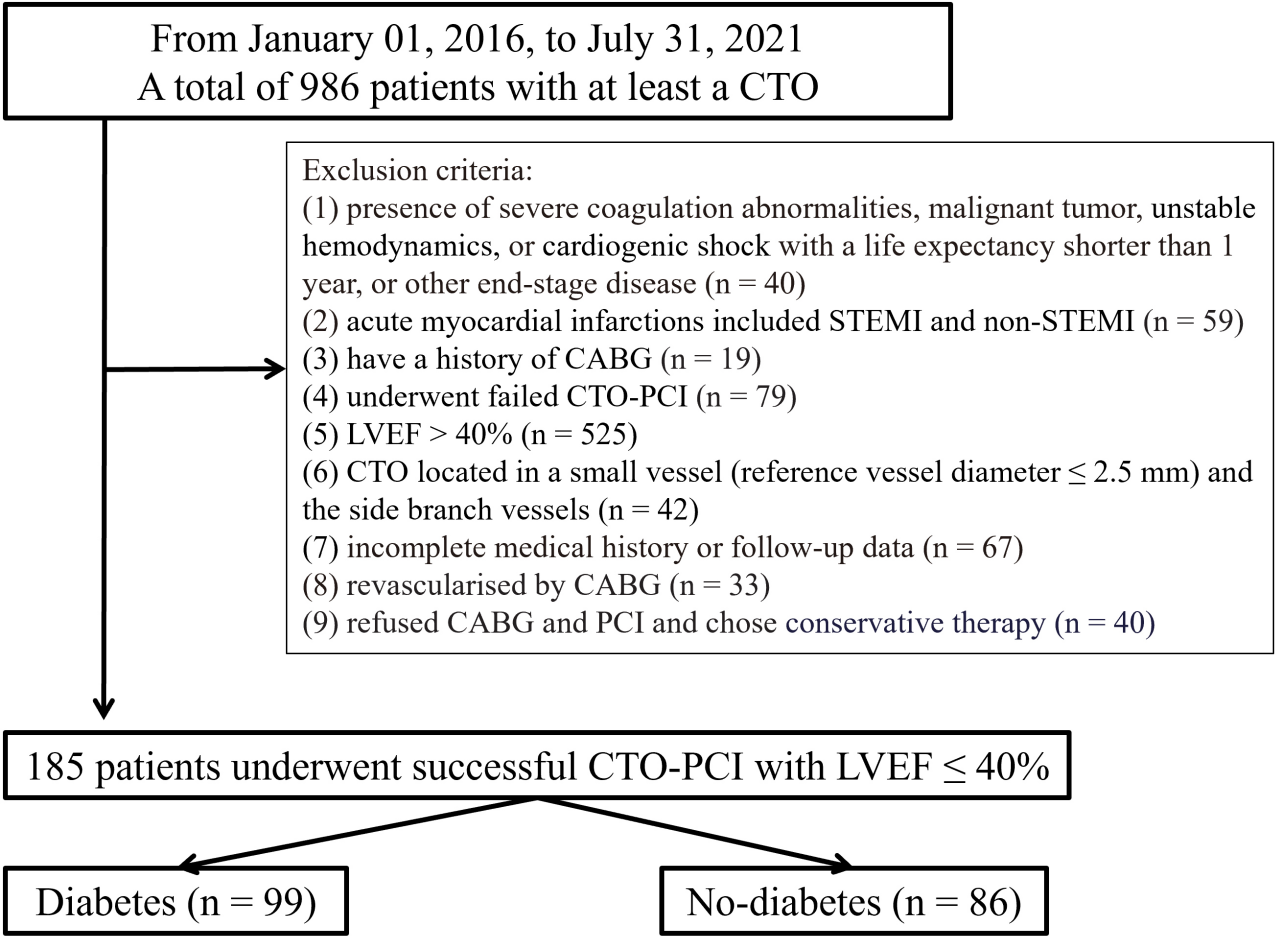
**Study flowchart**. CABG, coronary artery bypass grafting; CTO, chronic total occlusion; PCI, percutaneous coronary intervention; STEMI, ST-segment elevation myocardial infarction; LVEF, left ventricular ejection fraction.

This single-center, observational, retrospective study was conducted in Xiangtan Central Hospital, which included patients who underwent PCI for CTO between January 1, 2016, and July 31, 2021. The inclusion criteria of the study were that the patient had to have at least one CTO in a main coronary artery, stable vital signs, a LVEF of 40% or less, and a successful revascularization via PCI. All patients who received the treatment for CTO revascularization had symptoms suggestive of stable angina and/or noninvasive imaging for functional ischemia preference. The criteria for exclusion included (1) severe coagulation abnormalities, malignant tumors, unstable hemodynamics, cardiogenic shock with a projected life span of less than one year, or other terminal conditions; (2) acute myocardial infarctions included ST-segment elevation myocardial infarction (STEMI) and non-STEMI; (3) a previous history of coronary artery bypass grafting (CABG); (4) unsuccessful CTO-PCI; (5) an LVEF >40%; (6) CTO was located in a small vessel (reference vessel diameter ≤2.5 mm) and its side branch vessels; (7) lack of medical records or additional details; (8) revascularized by CABG; (9) refused CABG and PCI and chose conservative therapy (Fig. 1). The Ethics Committee of Xiangtan Central Hospital approved the research, which followed the principles outlined in the updated 2013 Declaration of Helsinki. Each participant provided informed consent before their participation (X201781019-1).

### 2.2 Definitions

A diagnosis of CTO was made when angiographic evidence revealed a thrombolysis in myocardial infarction (TIMI) flow grade of 0 in an occluded artery segment that was present for more than three months [16]. We classified non-CTO lesions as having a stenosis diameter of 50% for the left main (LM) artery and 70% for non-LM CAD within vessels with a diameter of at least 2.5 mm [17]. DM was identified by the administration of oral hypoglycemic agents or insulin, a fasting plasma glucose level of ≥7.0 mmol/L (126 mg/dL), or a 2-hour plasma glucose level of ≥11.1 mmol/L (200 mg/dL) after a standard 75-gram oral glucose tolerance test [18]. Multicenter chronic total occlusion registry in Japan (J-CTO) is a multicenter registry in Japan [19] that focuses on CTO cases. We recommended revascularization in cases where angiography revealed a severe narrowing of the vessels with a decrease in diameter of at least 70% and where a fractional flow reserve of less than 0.80 indicated a significant loss in blood flow. We defined complete revascularization as the process of treating all major stenoses in the coronary epicardial vessels within the same hospital stay [20]. The definition of technical success for CTO-PCI involved achieving blood flow through the blocked artery with a TIMI flow grade of 3 and a residual narrowing of less than 30% [16]. In patients with chronic kidney disease (CKD), their estimated glomerular filtration rate (eGFR) had to be less than 60 mL/min/1.73 m^2^ for a minimum of three months, or they required chronic dialysis [21]. Two-dimensional transthoracic echocardiography was used to measure the LVEF using the biplane Simpson’s method [22]. A cutoff value of 40% for LVEF was used to identify individuals with LV systolic dysfunction, consistent with previous clinical trials [23]. Patients were diagnosed with systolic LV failure when echocardiographic data revealed an ejection fraction (EF) of 40% or less and were receiving optimal medical therapy, as recommended by the guidelines [24].

### 2.3 PCI Procedure and Medical Treatments

We selectively performed PCI on symptomatic patients suffering from non-CTO lesions. For the CTO-PCI procedure, standard methods and guidelines were used, including bilateral injections, the hybrid algorithm, tapered-tip guidewires, stiff wires, parallel wires, microcatheters, and the retrograde method upon availability [25]. After a previous balloon angioplasty procedure, drug-eluting stents were inserted, and anticoagulant medication was administered during the PCI. Dual antiplatelet therapy for a minimum of one year and cardiovascular drugs, such as beta-blockers, calcium channel blockers, inhibitors of the renin–angiotensin–aldosterone inhibitors system, and statins, was also administered during the follow-up period.

### 2.4 Clinical Outcomes and Follow-Up 

MACEs were defined as cardiac death, myocardial infarction, target vessel revascularization, and all-cause mortality [26]. Meanwhile, in-hospital MACEs, including the above clinical adverse events, were assessed before hospital discharge. The primary endpoint was a MACE, with cardiac mortality as the secondary endpoint. Patients were evaluated at one-, six-, and twelve months post-PCI and annually after that for a maximum of 24 months through hospital record reviews, telephone interviews, and outpatient visits conducted by research coordinators.

### 2.5 Statistical Analysis

The categorical variables were analyzed using either the Chi-square or Fisher’s exact tests, with results presented as frequencies and percentages. Continuous variables were expressed as the mean ± standard deviation. They were compared between cohorts employing Student’s *t*-test. We calculated the cumulative survival incidence without adverse events using Kaplan–Meier analysis and evaluated it using log-rank testing. Stepwise variable selection was employed to create multi-variable Cox proportional hazard models, with the entrance and exit criteria set at a significance level of *p* ≤ 0.1. We calculated the hazard ratios (HRs) and their corresponding 95% confidence intervals (95% CIs). A statistically significant result was defined as a two-sided *p*-value of below 0.05. SPSS 26.0 tool (IBM Corp., Armonk, NY, USA) was used for the analyses.

## 3. Results

### 3.1 Baseline Clinical Characteristics 

A total of 185 patients underwent successful PCI for CTO, of which 99 (53.5%) had DM. The clinical features of patients are listed in Table 1. Chronic kidney disease was significantly increased in patients with DM compared to those without DM (29.3 vs. 18.6%, *p* < 0.001). DM patients also had higher serum creatinine (147.2 ± 28.0 vs. 93.8 ± 18.6 µmol/L, *p* < 0.001) and hemoglobin A1c (7.9 ± 1.8 vs. 5.8 ± 0.4, *p* < 0.001) and lower eGFR (66.9 ± 21.0 vs. 83.2 ± 25.0 mL/min/1.73 m^2^, *p* < 0.001) than patients without DM. In addition, DM individuals had a lower incidence of angiotensin-converting enzyme inhibitors (24.2 vs. 54.7%, *p* < 0.001) and angiotensin receptor blockers (18.2 vs. 29.1%, *p* = 0.010) compared to those without DM.

**Table 1.** **Baseline characteristics**.

Characteristic		DM (n = 99)	Non-DM (n = 86)	*p*-value
Males, n (%)		82 (82.8)	69 (80.2)	0.551
Age, y		62.3 ± 11.1	63.2 ± 12.4	0.546
BMI, kg/m^2^		27.1 ± 3.7	26.6 ± 2.3	0.204
Smoker or previous smoker, n (%)		50 (50.5)	43 (50.0)	0.945
Hypertension, n (%)		77 (77.8)	60 (70.0)	0.189
Cerebrovascular disease, n (%)		26 (26.3)	19 (22.1)	0.583
Peripheral artery disease, n (%)		18 (18.2)	19 (22.1)	0.441
Previous MI, n (%)		26 (26.3)	24 (27.9)	0.609
Previous PCI, n (%)		18 (18.2)	20 (23.3)	0.340
Chronic pulmonary disease, n (%)		4 (4.0)	5 (5.8)	0.737
Family history of CHD, n (%)		6 (6.1)	7 (8.1)	0.617
Chronic kidney disease, n (%)		29 (29.3)	16 (18.6)	<0.001
CCS score, n (%)				0.384
	I	6 (6.1)	7 (8.1)	
	II	18 (18.2)	11 (12.8)	
	III	26 (26.3)	26 (30.2)	
	IV	18 (18.2)	21 (24.4)	
NYHA functional class, n (%)				0.271
	I	27 (27.3)	22 (25.6)	
	II	45 (45.5)	41 (47.7)	
	III	21 (21.2)	19 (22.0)	
	IV	6 (6.1)	4 (4.7)	
LVEF at baseline		36.2 ± 4.7	37.1 ± 2.50	0.772
LVEF after PCI		44.2 ± 5.84	46.2 ± 6.73	0.361
ICD		15 (15.2)	16 (18.6)	0.411
Homocysteine, µmol/L		18.0 ± 11.5	14.0 ± 3.9	0.315
Lactate, mmol/L		2.3 ± 0.6	2.6 ± 0.4	0.220
Serum creatinine, µmol/L		147.2 ± 28.0	93.8 ± 18.6	<0.001
eGFR, mL/min/1.73 m^2^		66.9 ± 21.0	83.2 ± 25.0	<0.001
Hemoglobin A1c		7.9 ± 1.8	5.8 ± 0.4	<0.001
Total cholesterol, mmol/L		4.1 ± 1.2	4.0 ± 1.3	0.557
Triglycerides, mmol/L		2.2 ± 2.0	2.1 ± 0.7	0.312
HDL-c, mmol/L		0.9 ± 0.2	0.9 ± 0.3	0.400
LDL-c, mmol/L		2.5 ± 0.9	2.5 ± 1.1	0.997
Drug treatment				
	Aspirin, n (%)	99 (100)	86 (100)	1.000
	Clopidogre, n (%)	98 (99.0)	84 (97.7)	0.962
	Ticagrelor, n (%)	1 (1.0)	2 (2.3)	0.901
	ACEI, n (%)	24 (24.2)	47 (54.7)	<0.001
	ARB, n (%)	18 (18.2)	25 (29.1)	0.010
	Beta-blocker, n (%)	82 (82.8)	70 (81.4)	0.566
	Calcium channel blocker, n (%)	25 (25.3)	27 (31.4)	0.408
	Diuretic, n (%)	51 (51.5)	46 (53.5)	0.753
	Nitrate, n (%)	34 (34.3)	25 (29.1)	0.383
	Statin, n (%)	94 (94.9)	84 (97.7)	0.580

BMI, body mass index; MI, myocardial infarction; CHD, coronary atherosclerotic heart disease; CCS, Canadian Cardiovascular Society; NYHA, New York Heart Association; PCI, percutaneous coronary intervention; LVEF, left ventricular ejection fraction; ICD, implantable cardioverter defibrillator; eGFR, estimated glomerular filtration rate; HDL-c, high-density lipoprotein cholesterol; LDL-c, low-density lipoprotein cholesterol; ACEI, angiotensin-converting enzyme inhibitors; ARB, angiotensin receptor blocker; DM, diabetes mellitus.

### 3.2 Angiographic and Procedural Characteristics

Table 2 displays the angiographic details and procedure features. Patients with DM exhibited a significantly higher prevalence of multi-vessel disease compared to those with no DM (85.9% vs. 73.3%, *p* = 0.017). Patients with DM exhibited a greater number of lesions per patient (2.61 ± 0.93 vs. 2.20 ± 0.98, *p* = 0.001) and higher J-CTO scores (2.48 ± 1.13 vs. 2.01 ± 1.15, *p* = 0.015) and Synergy between Percutaneous Coronary Intervention with Taxus and Cardiac Surgery (SYNTAX) scores (23.64 ± 8.71 vs. 21.17 ± 8.33, *p* = 0.041). The rates of complete revascularization were comparable between patients with DM (82.8%) and those without DM (86.0%).

**Table 2.** **Angiographical characteristics and procedural details**.

Characteristic		DM (n = 99)	Non-DM (n = 86)	*p*-value
Vascular lesion, n (%)				
	LM	14 (14.1)	10 (11.6)	0.571
	LAD	81 (81.8)	65 (75.6)	0.189
	LCX	57 (57.6)	52 (60.5)	0.206
	RCA	76 (76.8)	65 (75.6)	0.645
Non-CTO target vessel, n (%)				
	LM	10 (10.1)	8 (8.1)	0.303
	LAD	61 (61.6)	59 (68.6)	0.792
	LCX	29 (29.3)	23 (26.7)	0.622
	RCA	55 (55.6)	49 (57.0)	0.814
Multi-vessel disease, n (%)		85 (85.9)	63 (73.3)	0.017
Number of lesions per patient		2.61 ± 0.93	2.20 ± 0.98	0.001
Vessels with CTO				
	LAD	32 (32.3)	35 (35.4)	0.848
	LCX	36 (36.4)	28 (32.6)	0.510
	RCA	53 (53.5)	38 (44.2)	0.055
Multi-CTO lesion, n (%)		29 (29.3)	24 (27.9)	0.631
Number of CTO per patient		1.40 ± 0.61	1.34 ± 0.56	0.087
Location of CTO				
	Proximal	55 (55.6)	48 (55.8)	0.806
	Mid	43 (43.4)	33 (38.4)	0.690
	Distal	23 (23.2)	20 (23.2)	0.995
Ostial location, n (%)		10 (10.1)	8 (9.3)	0.727
In-stent occlusion, n (%)		7 (7.1)	10 (11.6)	0.069
Lesion length, mm		28.39 ± 16.96	27.85 ± 20.57	0.672
Lesion length ≥20 mm, n (%)		68 (68.7)	55 (64.0)	0.130
Blunt stump, n (%)		66 (66.7)	60 (70.0)	0.169
Tortuosity ≥45°, n (%)		28 (28.3)	24 (27.9)	0.801
Calcification, n (%)		32 (32.3)	25 (29.1)	0.693
Reattempt, n (%)		9 (9.1)	8 (9.3)	0.723
J-CTO score		2.48 ± 1.13	2.01 ± 1.15	0.015
SYNTAX score		23.64 ± 8.71	21.17 ± 8.33	0.041
Complete revascularization		79 (79.8)	74 (86.0)	0.077
Success of PCI for CTO		84 (84.8)	78 (90.7)	0.151
Contrast amount, mL		250 ± 180	240 ± 100	0.473
Procedural time, min		119.14 ± 69.29	117.21 ± 69.97	0.667

DM, diabetes mellitus; LAD, left anterior descending artery; LCX, left circumflex artery; LM, left main artery; CTO chronic total occlusion; J-CTO, multicenter CTO registry in Japan; RCA, right coronary artery; PCI, percutaneous coronary intervention; SYNTAX, Synergy between Percutaneous Coronary Intervention with Taxus and Cardiac Surgery.

### 3.3 In-Hospital and 2-Year Total Clinical Outcomes

**Fig. 2.**
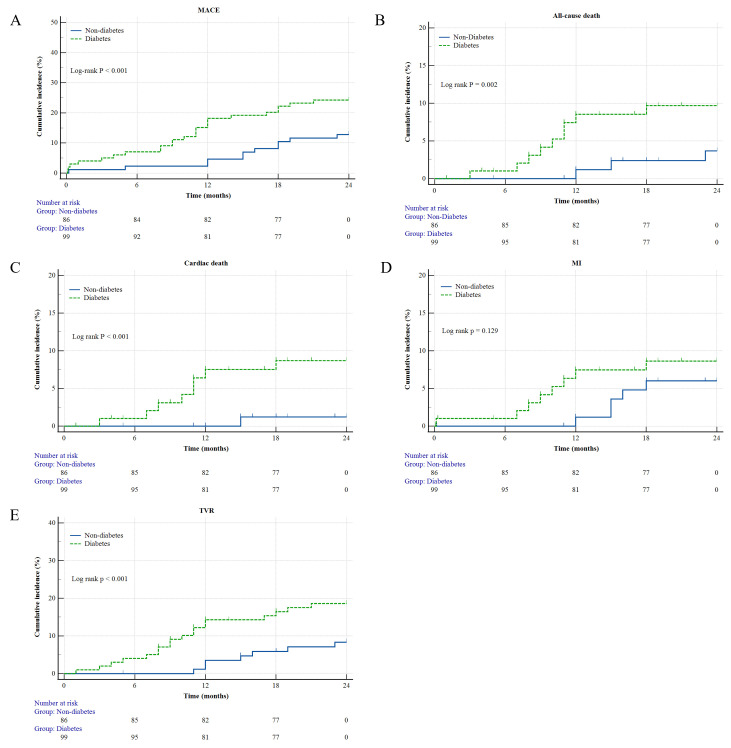
**Kaplan–Meier survival curves for 2 years**. (A) MACEs. (B) All-cause death. (C) Cardiac death. (D) MI. (E) TVR. MACEs, major adverse cardiac events; MI, myocardial infarction; TVR, target vessel revascularization.

Table 3 shows the clinical outcomes between patients with and without DM in-hospital and two years. No significant differences in in-hospital MACEs were detected between the DM and non-DM groups. During the two-year follow-up, 35 (18.9%) patients experienced MACEs, and death occurred in 12 patients (6.5%). The DM group had higher incidences of MACEs (24.2 vs. 12.8%, *p* < 0.001), all-cause mortality (9.1 vs. 3.5%, *p* = 0.002), cardiac death (8.1 vs. 1.2%, *p* < 0.001), and target vessel revascularization (TVR) (18.2 vs. 7.1%, *p* < 0.001). Kaplan–Meier curve analysis corroborated these findings (Fig. 2).

**Table 3.** **In-hospital and 2-year total clinical outcomes for patients with DM and those without DM**.

Characteristic	DM (n = 99)	Non-DM (n = 86)	*p*-value
In-hospital MACEs	1 (1.0)	3 (3.5)	0.300
All-cause death	0	0	-
Cardiac death	0	0	-
Myocardial infarction	3 (3.5)	1 (1.0)	0.300
Target vessel revascularization	0	0	-
2-year total MACEs	24 (24.2)	11 (12.8)	<0.001
All-cause death	9 (9.1)	3 (3.5)	0.002
Cardiac death	8 (8.1)	1 (1.2)	<0.001
Myocardial infarction	8 (8.1)	5 (5.8)	0.129
Target vessel revascularization	18 (18.2)	7 (7.1)	<0.001

DM, diabetes mellitus; MACEs, major adverse cardiac and cerebrovascular events.

### 3.4 Independent Predictors for MACEs

The univariate and multivariate regression analyses in Table 4 indicated that the presence of DM is not an independent predictor of MACEs (HR: 0.58; 95% CI: 0.32 to 1.03; *p* = 0.260); meanwhile, multi-vessel disease (HR: 1.69; 95% CI: 1.21 to 2.36; *p* = 0.002) and CKD (HR: 1.38; 95% CI: 1.08 to 1.78; *p* = 0.011) were independent predictors of MACEs and complete revascularization was associated with a reduced incidence of MACEs (HR: 0.36; 95% CI: 0.14 to 0.88; *p* = 0.026).

**Table 4.** **Univariate and multivariate analyses for predictors of MACEs**.

		HR (95% CI)	*p*-value
Univariate			
	Age (per year increment)	0.98 (0.95–1.01)	0.296
	Male	0.99 (0.93–1.06)	0.863
	Hypercholesterolemia	0.69 (0.31–1.34)	0.261
	Hypertension	0.98 (0.56–1.77)	0.968
	The presence of DM	1.88 (1.09–3.28)	0.024
	Smoking	1.40 (0.81–2.41)	0.237
	CKD	3.59 (1.89–6.84)	<0.001
	LVEF at baseline	0.85 (0.42–1.62)	0.621
	Prior MI	1.99 (0.26–12.2)	0.475
	Multi-vessel disease	2.26 (1.33–3.91)	0.003
	CTO target vessel	1.11 (0.39–2.83)	0.838
	RCA	4.96 (2.18–11.8)	<0.001
	LAD	0.61 (0.14–1.95)	0.437
	LCX	1.18 (0.68–2.04)	0.552
	Total CTO length (mm)	1.95 (0.85–4.30)	0.117
	SYNTAX score	1.68 (0.50–5.05)	0.387
	J-CTO score	4.48 (1.28–28.3)	0.020
	Complete revascularization	0.29 (0.108–0.755)	0.012
	Blunt stump	1.64 (0.73–3.54)	0.231
	Tortuosity ≥45°	1.20 (0.48–2.77)	0.689
	Calcification	1.08 (0.43–2.46)	0.864
	Reattempt	0.82 (0.31–1.92)	0.662
Multivariate			
	CKD	1.38 (1.08–1.78)	0.011
	The presence of DM	0.58 (0.32–1.03)	0.260
	CTO vessel (RCA)	1.16 (0.11–11.53)	0.893
	J-CTO score	3.41 (0.20–16.88)	0.341
	Multi-vessel disease	1.69 (1.21–2.36)	0.002
	Complete revascularization	0.36 (0.14–0.88)	0.026

DM, diabetes mellitus; MI, myocardial infarction; MACEs, major adverse cardiac and cerebrovascular events; CTO, chronic total occlusion; LAD, left anterior descending coronary artery; LCX, left circumflex coronary artery; RCA, right coronary artery; J-CTO, multicenter CTO registry in Japan; CKD, chronic kidney disease; HR, hazard ratio; 95% CI, 95% confidence interval; LVEF, left ventricular ejection fraction; SYNTAX, Synergy between Percutaneous Coronary Intervention with Taxus and Cardiac Surgery.

## 4. Discussion

This is the first study to assess the clinical outcomes after a successful PCI for CTO in patients with and without DM who have LV systolic dysfunction over two years. The results revealed several key findings: (1) the presence of DM is not an independent risk factor for MACEs. In addition, multi-vessel disease and CKD were independent predictors of MACEs, whereas complete revascularization was associated with a decreased incidence of MACEs. (2) patients with DM had an increased incidence of CKD, higher serum creatinine and hemoglobin A1c levels, and lower eGFR. Patients with DM were less likely to utilize angiotensin-converting enzyme inhibitors medications and angiotensin receptor blockers than those without DM. (3) patients with DM exhibited an increased incidence of multi-vessel CAD, a greater number of lesions per patient, and elevated J-CTO and SYNTAX scores than non-DM patients. (4) DM was associated with decreased long-term survival benefits regarding MACEs following a successful CTO-PCI.

### 4.1 CTO and DM

In this study, 53.5% (99 out of 185) of patients with LV systolic dysfunction undergoing CTO-PCI were diagnosed with DM. This finding aligns with previous studies reporting a 27–45% DM prevalence among CTO patients [5, 7, 8]. It has been consistently observed that patients with DM exhibit a more accelerated progression of atherosclerotic burden and more widespread coronary atherosclerosis compared to non-DM individuals [4, 27]. In addition, previous research has shown that DM patients are more likely to experience hypertension, previous PCIs, and strokes [10]. Our findings further indicate that DM patients have higher rates of chronic kidney disease, elevated serum creatinine and hemoglobin A1c levels, and reduced eGFR. Patients with DM are more prone to have complex and severe CAD. This condition frequently involves the presence of multi-vessel disease, diffuse narrowing, and calcifications in the coronary arteries [28]. This study also revealed that diabetic patients more often present with multi-vessel disease, higher J-CTO scores, and SYNTAX scores compared to non-diabetic patients, as reported in earlier studies [5, 8, 10]. In patients suffering from complex or multi-vessel CAD who have PCI, the occurrence of CTO was demonstrated to have a negative impact on the extent of revascularization and long-term clinical outcomes, including death, repeat revascularization, and stent thrombosis [29]. This impact is notably more pronounced in DM patients, who exhibit a two-fold higher prevalence of CTOs than non-diabetic individuals [30]. Furthermore, while DM significantly worsens the prognosis in CAD patients undergoing coronary revascularization, other cardiovascular risk factors, and comorbidities that adversely affect outcomes are also more prevalent in this group [31]. As a result, diabetic patients who have CTO-PCI have a greater cardiovascular risk profile compared to those without diabetes [6].

### 4.2 Clinical Outcomes 

Patients suffering from DM had a higher incidence of CTOs [30]. However, diabetic patients undergo PCI for CTO less often compared to non-diabetic patients [7, 32]. This phenomenon, known as the treatment-risk paradox, reflects the less frequent intervention in high-risk compared to lower-risk patients within the PCI population [4]. Patients with diabetes continue to have an increased risk of long-term MACEs following PCI, even with the use of newer-generation drug-eluting stents. This is mainly because they are more likely to require repeat procedures to reopen the blocked blood vessels, regardless of the severity of their underlying CAD [33]. Guo *et al*. [8] revealed a significant increase in MACEs among diabetes patients after a successful PCI for CTO. Similarly, Sanguineti F *et al*. [9] reported elevated MACE rates in diabetic CTO patients during a 4.2-year follow-up period. Systematic reviews have consistently indicated that diabetic patients undergoing successful CTO-PCI have an increased risk of adverse clinical outcomes compared to non-diabetic patients [34, 35]. Zhu *et al*. [36] found that diabetic patients experienced higher rates of MACEs following successful CTO-PCI, particularly within follow-up periods shorter than three years. Wang *et al*. [37], analyzing 5-year outcomes in 719 patients post-successful CTO-PCI (316 diabetic and 403 non-diabetic), noted that non-diabetic patients showed superior long-term survival benefits in terms of MACEs contrasted with diabetic patients. However, in several studies, there were no statistically significant variations in MACE rates involving diabetic and non-diabetic patients over follow-up periods ranging from 1.7 to 5 years [11, 12, 13]. In these present studies, MACEs were consistently increased in diabetic patients compared to non-diabetic patients following successful CTO-PCI.

Patients with DM are more prone to developing cardiomyopathy associated with LV systolic dysfunction, potentially leading to significant decreases in the viability of myocardium downstream from a CTO [9, 38]. Furthermore, the presence of a CTO in a myocardial infarction-affected artery often results in a significant scar and, notably, a larger area surrounding the scar [39]. This larger border zone is associated with an increased incidence of arrhythmias and sudden cardiac death [39]. Revascularizing the viable or ischemic myocardium in the CTO region can improve survival by reducing scar tissue formation after a heart attack [39]. Our research sample found no significant disparities in the rates of complete revascularization between the diabetic and non-diabetic cohorts. A Study has shown that performing complete myocardial revascularization is associated with lower mortality rates, a reduced incidence of MI, and a decreased need for repeat revascularization [40]. DM patients who undergo incomplete revascularization are at a higher risk of long-term MACEs, including death, MI, stroke, or the need for repeat revascularization [41]. Our study indicates that LV systolic dysfunction patients with DM may experience more unfavorable long-term clinical outcomes following successful CTO PCI because of multiple contributing factors. DM, which is a well-known risk factor for CAD, is associated with more negative angiographic and clinical features [6, 31]. Moreover, patients with DM typically present with an increased number of lesions per patient, potentially increasing the risk of adverse outcomes [42]. Secondly, DM may exacerbate the risk of adverse outcomes by negatively affecting post-procedure blood glucose levels, lipid metabolism, insulin resistance, susceptibility to coronary plaque formation, and vascular endothelial function [43, 44]. The increased amount of platelet aggregation among DM patients and hypo-responsiveness to anti-platelet drugs such as aspirin and clopidogrel result in increased adverse outcomes. The impaired coronary collateralization seen in DM patients might also play a role in their unfavorable prognosis [45].

### 4.3 Predictors of MACEs

Consistent with previous studies, our findings corroborate that multi-vessel disease is linked to increased risks of MACEs [35]. Although the prevalence of CTOs was comparable across coronary territories between the two groups, in our CTO cohort, diabetic patients exhibited a higher rate of multi-vessel disease, which could predispose them to elevated long-term mortality and MACEs. Previous studies also found a strong link between CKD and a higher frequency of MACEs. Previous studies have identified renal insufficiency as a strong independent predictor of poor clinical outcomes post-PCI for CTO [11, 13]. The influence of CKD on patients with CTO requires further investigation to determine whether the observed adverse outcomes are attributable solely to the additional risk posed by CKD in conjunction with DM or if CKD alone constitutes an independent risk variable that contributes to negative cardiovascular findings within patients suffering from CTO lesions. In our research, complete revascularization was independently associated with a decreased risk of MACEs. Our results suggest that complete revascularization might mitigate future coronary events by alleviating the burden on non-CTO arteries in patients with DM. This is because the myocardial territory supplied by a CTO artery receives collateral blood flow from other coronary arteries [46]. Our results indicate that all patients exhibited multi-vessel disease, involving at least half of the coronary system. Therefore, complete revascularization could alleviate the functional burden on non-CTO arteries. Moreover, in the event of subsequent coronary incidents, the revascularized CTO artery could potentially support the affected artery [47, 48]. Similar to the results of previous studies [11, 13], our research has shown that DM was not an independent predictor for MACEs. This result may be due to the evolution of equipment, new application techniques, and the accumulation of recent CTO-PCI experience.

## 5. Limitation

This research has several limitations. First, its retrospective design may introduce selection and information biases. Second, the research findings, performed at a single center, might not be generalizable to a wider population. It is imperative, therefore, to validate these results through multi-center clinical trials. Third, the limited sample size may impede the research’s ability to identify substantial disparities between the cohorts. Fourth, the absence of a medically treated comparison group precluded a comprehensive outcome comparison. Only successful CTO PCI cases were included, excluding failed procedures, which could affect the validity of the conclusions. Fifth, data collection relied on hospital information systems and telephone follow-ups, potentially introducing unknown confounding factors that could skew the results. Specific data, such as coronary collateral scoring and glycemic control during the extended follow-up, were lacking, possibly compromising the precise assessment of future adverse event risks in CTO patients. Additionally, the selection criteria could also influence the long-term outcome, unmatched baseline characteristics (such as the rate of multi-vessel coronary disease, complete revascularization and CKD and baseline renal function and SYNTAX and J-CTO score), and treatment choice (such as angiotensin-converting enzyme inhibitors and angiotensin receptor blocker). Finally, 19 patients had a history of CABG, and 15 patients rejected PCI for main coronary artery CTO and chose optimal medical therapy. The other four patients chose revascularization for left internal thoracic artery graft or saphenous vein graft and rejected PCI for main coronary artery CTO. Thus, the present research excluded post-CABG patients.

## 6. Conclusions

Our registry found that LV systolic dysfunction patients with DM who underwent successful CTO revascularization had increased rates of MACEs over a two-year follow-up compared to patients without DM. However, the presence of DM is not an independent risk factor for MACEs. Moreover, multi-vessel disease and CKD significantly increased the incidence of MACEs, whereas complete revascularization was associated with a reduced risk. Further large-scale, rigorously designed, randomized controlled trials with extended follow-ups are essential to corroborate these findings.

## Availability of Data and Materials

The datasets generated during and/or analyzed during the current study are available from the corresponding author on reasonable request.

## References

[1] Holmes DR, Barsness GW (2019). Percutaneous Coronary Intervention for Chronic Total Occlusions. *Circulation. Cardiovascular Interventions*.

[2] Guo L, Wu J, Zhong L, Ding H, Xu J, Zhou X (2020). Two-year clinical outcomes of medical therapy vs. revascularization for patients with coronary chronic total occlusion. *Hellenic Journal of Cardiology: HJC*.

[3] Yan Y, Zhang M, Yuan F, Liu H, Wu D, Fan Y (2019). Successful revascularization versus medical therapy in diabetic patients with stable right coronary artery chronic total occlusion: a retrospective cohort study. *Cardiovascular Diabetology*.

[4] Roffi M, Angiolillo DJ, Kappetein AP (2011). Current concepts on coronary revascularization in diabetic patients. *European Heart Journal*.

[5] Choi KH, Yang JH, Song YB, Hahn JY, Choi JH, Gwon HC (2017). Long-term clinical outcomes of patients with coronary chronic total occlusion treated with percutaneous coronary intervention versus medical therapy according to presence of diabetes mellitus. *EuroIntervention: Journal of EuroPCR in Collaboration with the Working Group on Interventional Cardiology of the European Society of Cardiology*.

[6] Salisbury AC, Sapontis J, Grantham JA, Qintar M, Gosch KL, Lombardi W (2017). Outcomes of Chronic Total Occlusion Percutaneous Coronary Intervention in Patients With Diabetes: Insights From the OPEN CTO Registry. *JACC. Cardiovascular Interventions*.

[7] Fefer P, Knudtson ML, Cheema AN, Galbraith PD, Osherov AB, Yalonetsky S (2012). Current perspectives on coronary chronic total occlusions: the Canadian Multicenter Chronic Total Occlusions Registry. *Journal of the American College of Cardiology*.

[8] Guo L, Wang J, Ding H, Meng S, Zhang X, Lv H (2020). Long-term outcomes of medical therapy versus successful recanalisation for coronary chronic total occlusions in patients with and without type 2 diabetes mellitus. *Cardiovascular Diabetology*.

[9] Sanguineti F, Garot P, O’Connor S, Watanabe Y, Spaziano M, Lefèvre T (2017). Chronic total coronary occlusion treated by percutaneous coronary intervention: long-term outcome in patients with and without diabetes. *EuroIntervention: Journal of EuroPCR in Collaboration with the Working Group on Interventional Cardiology of the European Society of Cardiology*.

[10] Zhao S, Chen Y, Wang Q, Zhu B, Wei Z, Wang Z (2022). Benefits of successful percutaneous coronary intervention in chronic total occlusion patients with diabetes. *Cardiovascular Diabetology*.

[11] Fu D, Li H, Gao T, Liu M, Feng L, Li C (2021). Comparison of long-term clinical outcomes of percutaneous coronary intervention for chronic total occlusion between patients with and without diabetes mellitus: a single-center retrospective observational study. *Annals of Palliative Medicine*.

[12] Tsai CT, Huang WC, Teng HI, Tsai YL, Lu TM (2020). Long term clinical impact of successful recanalization of chronic total occlusion in patients with and without type 2 diabetes mellitus. *Cardiovascular Diabetology*.

[13] Mashaly A, Rha SW, Choi BG, Baek MJ, Ryu YG, Choi SY (2018). Impact of diabetes mellitus on 5-year clinical outcomes in patients with chronic total occlusion lesions. *Coronary Artery Disease*.

[14] Galassi AR, Boukhris M, Toma A, Elhadj ZI, Laroussi L, Gaemperli O (2017). Percutaneous Coronary Intervention of Chronic Total Occlusions in Patients With Low Left Ventricular Ejection Fraction. *JACC. Cardiovascular Interventions*.

[15] Sengodan P, Davies RE, Matsuno S, Chan AK, Kearney K, Salisbury A (2023). Chronic Total Occlusion Interventions in Patients with Reduced Ejection Fraction. *Current Cardiology Reports*.

[16] Stone GW, Kandzari DE, Mehran R, Colombo A, Schwartz RS, Bailey S (2005). Percutaneous recanalization of chronically occluded coronary arteries: a consensus document: part I. *Circulation*.

[17] Lee SW, Lee PH, Ahn JM, Park DW, Yun SC, Han S (2019). Randomized Trial Evaluating Percutaneous Coronary Intervention for the Treatment of Chronic Total Occlusion. *Circulation*.

[18] Alberti KG, Zimmet PZ (1998). Definition, diagnosis and classification of diabetes mellitus and its complications. Part 1: diagnosis and classification of diabetes mellitus provisional report of a WHO consultation. *Diabetic Medicine: a Journal of the British Diabetic Association*.

[19] Morino Y, Abe M, Morimoto T, Kimura T, Hayashi Y, Muramatsu T (2011). Predicting successful guidewire crossing through chronic total occlusion of native coronary lesions within 30 minutes: the J-CTO (Multicenter CTO Registry in Japan) score as a difficulty grading and time assessment tool. *JACC. Cardiovascular Interventions*.

[20] Di Mario C, Werner GS, Sianos G, Galassi AR, Büttner J, Dudek D (2007). European perspective in the recanalisation of Chronic Total Occlusions (CTO): consensus document from the EuroCTO Club. *EuroIntervention: Journal of EuroPCR in Collaboration with the Working Group on Interventional Cardiology of the European Society of Cardiology*.

[21] National Kidney Foundation (2002). K/DOQI clinical practice guidelines for chronic kidney disease: evaluation, classification, and stratification. *American Journal of Kidney Diseases: the Official Journal of the National Kidney Foundation*.

[22] Lang RM, Badano LP, Mor-Avi V, Afilalo J, Armstrong A, Ernande L (2015). Recommendations for cardiac chamber quantification by echocardiography in adults: an update from the American Society of Echocardiography and the European Association of Cardiovascular Imaging. *European Heart Journal. Cardiovascular Imaging*.

[23] Daneault B, Généreux P, Kirtane AJ, Witzenbichler B, Guagliumi G, Paradis JM (2013). Comparison of Three-year outcomes after primary percutaneous coronary intervention in patients with left ventricular ejection fraction <40% versus ≥ 40% (from the HORIZONS-AMI trial). *The American Journal of Cardiology*.

[24] Rousan TA, Thadani U (2019). Stable Angina Medical Therapy Management Guidelines: A Critical Review of Guidelines from the European Society of Cardiology and National Institute for Health and Care Excellence. *European Cardiology*.

[25] Levine GN, Bates ER, Blankenship JC, Bailey SR, Bittl JA, Cercek B (2011). 2011 ACCF/AHA/SCAI Guideline for Percutaneous Coronary Intervention: a report of the American College of Cardiology Foundation/American Heart Association Task Force on Practice Guidelines and the Society for Cardiovascular Angiography and Interventions. *Circulation*.

[26] Cutlip DE, Windecker S, Mehran R, Boam A, Cohen DJ, van Es GA (2007). Clinical end points in coronary stent trials: a case for standardized definitions. *Circulation*.

[27] Martín-Timón I, Sevillano-Collantes C, Segura-Galindo A, Del Cañizo-Gómez FJ (2014). Type 2 diabetes and cardiovascular disease: Have all risk factors the same strength?. *World Journal of Diabetes*.

[28] Nicholls SJ, Tuzcu EM, Kalidindi S, Wolski K, Moon KW, Sipahi I (2008). Effect of diabetes on progression of coronary atherosclerosis and arterial remodeling: a pooled analysis of 5 intravascular ultrasound trials. *Journal of the American College of Cardiology*.

[29] Farooq V, Serruys PW, Garcia-Garcia HM, Zhang Y, Bourantas CV, Holmes DR (2013). The negative impact of incomplete angiographic revascularization on clinical outcomes and its association with total occlusions: the SYNTAX (Synergy Between Percutaneous Coronary Intervention with Taxus and Cardiac Surgery) trial. *Journal of the American College of Cardiology*.

[30] Ledru F, Ducimetière P, Battaglia S, Courbon D, Beverelli F, Guize L (2001). New diagnostic criteria for diabetes and coronary artery disease: insights from an angiographic study. *Journal of the American College of Cardiology*.

[31] Iglesias JF, Degrauwe S, Rigamonti F, Noble S, Roffi M (2019). Percutaneous Coronary Intervention of Chronic Total Occlusions in Patients with Diabetes Mellitus: a Treatment-Risk Paradox. *Current Cardiology Reports*.

[32] Grantham JA, Marso SP, Spertus J, House J, Holmes DR, Rutherford BD (2009). Chronic total occlusion angioplasty in the United States. *JACC. Cardiovascular Interventions*.

[33] Koskinas KC, Siontis GCM, Piccolo R, Franzone A, Haynes A, Rat-Wirtzler J (2016). Impact of Diabetic Status on Outcomes After Revascularization With Drug-Eluting Stents in Relation to Coronary Artery Disease Complexity: Patient-Level Pooled Analysis of 6081 Patients. *Circulation. Cardiovascular Interventions*.

[34] Guan J, Li X, Gong S, Li L (2022). Impact of diabetes mellitus on all and successful percutaneous coronary intervention outcomes for chronic total occlusions: A systematic review and meta-analysis. *Heart & Lung: the Journal of Critical Care*.

[35] Latif A, Ahsan MJ, Kabach A, Kapoor V, Mirza M, Ahsan MZ (2022). Impact of Diabetes Mellitus on Outcomes of Percutaneous Coronary Intervention in Chronic Total Occlusions: A Systematic Review and Meta-Analysis. *Cardiovascular Revascularization Medicine: Including Molecular Interventions*.

[36] Zhu Y, Meng S, Chen M, Liu K, Jia R, Li H (2021). Long-term prognosis of chronic total occlusion treated by successful percutaneous coronary intervention in patients with or without diabetes mellitus: a systematic review and meta-analysis. *Cardiovascular Diabetology*.

[37] Wang P, Yuan D, Jia S, Zhu P, Zhang C, Liu Y (2021). 5-Year Clinical Outcomes of Successful Recanalisation for Coronary Chronic Total Occlusions in Patients With or Without Type 2 Diabetes Mellitus. *Frontiers in Cardiovascular Medicine*.

[38] Damluji AA, Pomenti SF, Ramireddy A, Al-Damluji MS, Alfonso CE, Schob AH (2016). Influence of Total Coronary Occlusion on Clinical Outcomes (from the Bypass Angioplasty Revascularization Investigation 2 DiabetesTrial). *The American Journal of Cardiology*.

[39] Di Marco A, Paglino G, Oloriz T, Maccabelli G, Baratto F, Vergara P (2015). Impact of a chronic total occlusion in an infarct-related artery on the long-term outcome of ventricular tachycardia ablation. *Journal of Cardiovascular Electrophysiology*.

[40] Garcia S, Sandoval Y, Roukoz H, Adabag S, Canoniero M, Yannopoulos D (2013). Outcomes after complete versus incomplete revascularization of patients with multivessel coronary artery disease: a meta-analysis of 89,883 patients enrolled in randomized clinical trials and observational studies. *Journal of the American College of Cardiology*.

[41] Zimarino M, Ricci F, Romanello M, Di Nicola M, Corazzini A, De Caterina R (2016). Complete myocardial revascularization confers a larger clinical benefit when performed with state-of-the-art techniques in high-risk patients with multivessel coronary artery disease: A meta-analysis of randomized and observational studies. *Catheterization and Cardiovascular Interventions: Official Journal of the Society for Cardiac Angiography & Interventions*.

[42] Banning AP, Westaby S, Morice MC, Kappetein AP, Mohr FW, Berti S (2010). Diabetic and nondiabetic patients with left main and/or 3-vessel coronary artery disease: comparison of outcomes with cardiac surgery and paclitaxel-eluting stents. *Journal of the American College of Cardiology*.

[43] Du R, Zhang RY, Lu L, Shen Y, Pu LJ, Zhu ZB (2018). Increased glycated albumin and decreased esRAGE levels in serum are related to negative coronary artery remodeling in patients with type 2 diabetes: an Intravascular ultrasound study. *Cardiovascular Diabetology*.

[44] Yang ZK, Shen Y, Shen WF, Pu LJ, Meng H, Zhang RY (2015). Elevated glycated albumin and reduced endogenous secretory receptor for advanced glycation endproducts levels in serum predict major adverse cardio-cerebral events in patients with type 2 diabetes and stable coronary artery disease. *International Journal of Cardiology*.

[45] Shen Y, Ding FH, Dai Y, Wang XQ, Zhang RY, Lu L (2018). Reduced coronary collateralization in type 2 diabetic patients with chronic total occlusion. *Cardiovascular Diabetology*.

[46] Bucciarelli-Ducci C, Auger D, Di Mario C, Locca D, Petryka J, O’Hanlon R (2016). CMR Guidance for Recanalization of Coronary Chronic Total Occlusion. *JACC. Cardiovascular Imaging*.

[47] Rha SW, Li H, Choi CU, Choi BG (2022). Impact of complete revascularization on long-term clinical outcomes for patients with diabetes mellitus and coronary chronic total occlusion lesion. *Heart and Vessels*.

[48] Valenti R, Migliorini A, De Gregorio MG, Martone R, Berteotti M, Bernardini A (2020). Impact of complete percutaneous revascularization in elderly patients with chronic total occlusion. *Catheterization and Cardiovascular Interventions: Official Journal of the Society for Cardiac Angiography & Interventions*.

